# C_60_ fullerene against SARS-CoV-2 coronavirus: an in silico insight

**DOI:** 10.1038/s41598-021-97268-6

**Published:** 2021-09-07

**Authors:** Vasyl V. Hurmach, Maksim O. Platonov, Svitlana V. Prylutska, Peter Scharff, Yuriy I. Prylutskyy, Uwe Ritter

**Affiliations:** 1 Taras Shevchenko National University of Kyiv, Kyiv, 01601 Ukraine; 2 Institute of Molecular Biology and Genetics of NASU, Kyiv, 03143 Ukraine; 3 National University of Life and Environmental Science of Ukraine, Kyiv, 03041 Ukraine; 4 Institute of Chemistry and Biotechnology, Technical University of Ilmenau, 98693 Ilmenau, Germany

**Keywords:** Molecular medicine, Applied mathematics

## Abstract

Based on WHO reports the new SARS-CoV-2 coronavirus is currently widespread all over the world. So far > 162 million cases have been confirmed, including > 3 million deaths. Because of the pandemic still spreading across the globe the accomplishment of computational methods to find new potential mechanisms of virus inhibitions is necessary. According to the fact that C_60_ fullerene (a sphere-shaped molecule consisting of carbon) has shown inhibitory activity against various protein targets, here the analysis of the potential binding mechanism between SARS-CoV-2 proteins 3CLpro and RdRp with C_60_ fullerene was done; it has resulted in one and two possible binding mechanisms, respectively. In the case of 3CLpro, C_60_ fullerene interacts in the catalytic binding pocket. And for RdRp in the first model C_60_ fullerene blocks RNA synthesis pore and in the second one it prevents binding with Nsp8 co-factor (without this complex formation, RdRp can’t perform its initial functions). Then the molecular dynamics simulation confirmed the stability of created complexes. The obtained results might be a basis for other computational studies of 3CLPro and RdRp potential inhibition ways as well as the potential usage of C_60_ fullerene in the fight against COVID-19 disease.

## Introduction

Viral infections are widespread; they make up 95% of all known human infectious diseases. The coronaviruses are single-stranded RNA viruses that infect vertebrates. With the emergence of SARS-CoV-2 (severe acute respiratory syndrome-coronavirus-2), there are now few coronaviruses that are known to infect humans. Before COVID-19, only SARS-CoV (serve acute respiratory syndrome-CoV) and MERS-CoV (middle east respiratory syndrome-CoV) caused disease^1–4^. Therefore, anticoronaviral drug discovery has been a small effort relative to that for other viral diseases such as influenza. Given the rapid spread of COVID-19 and its relatively high mortality, filling the gap for coronavirus-specific drugs is urgent.

The SARS-CoV-2 genome comprises 11 ORFs (open reading frames). At the 5′-terminus ORF1a/b encoded polyprotein 1a and 1b which could be split into 16 different proteins; e. g. PLpro (papain-like protease), 3CLpro (chymotrypsin-like protease)/Mpro (main proteinase), Nsp9 (non-structural protein 9) binding protein, RdRp (RNA-dependent RNA polymerase), RNA helicase, exo-ribonuclease, endo-ribonuclease^5–8^. Contrary, 3′-terminus encoded proteins like S-glycoprotein, nucleocapsid protein, and others^9^. So, according to the above, isn’t surprising that the coronavirus life cycle comprises a number of potentially targetable steps, including endocytic entry into host cells (angiotensin-converting enzyme 2 (ACE2) and transmembrane protease serine 2 (TMPRSS2)), RNA replication, and transcription (helicase and RNA-dependent RNA polymerase (RdRp)), translation and proteolytic processing of viral proteins (3CLpro and PLpro), virion assembly, and release of new viruses through the exocytic systems^10^.

Amidst the various viral proteins, few are irreplaceable for the virus life cycle. 3CLpro protein has a crucial role in the replication and expression of viral genes^11,12^. The active site of this protein contains a catalytic dyad, where His 41 acts as general acid–base and Cys 145 acts as a nucleophile. The RdRp in complex with essential co-factors Nsp7 and Nsp8 is essential for virus RNA replication^13,14^. Based on recent studies, RdRp needs to create a complex with Nsp7 and Nsp8, such association activates the possibility to replicate long RNA molecules^14,15^. Thus, 3CLpro and RdRp appear to be promising protein targets for the development of inhibitors to treat SARS-Cov-2^4,16–22^.

The appearance of SARS-CoV-2 coronavirus with its real threat to human life requires the rapid development of innovative diagnostic tests and antiviral formulations. In this regards, nanobiomaterials represent alternative resource to fight coronaviruses at different stages of infection by selective action^23,24^. The main interest in carbon-based nanosystems lies precisely in their potential low toxicity^25,26^ and specific virus inhibition mechanisms^27^. One of the promising candidates for carbon antiviral nanoformulation can be C_60_ fullerene^27,28^. The highly symmetric nanostructure, such as C_60_ fullerene, has extraordinary geometric affinity with icosahedral viruses^29^. The creation of nanobiomaterials on the same scale and with similar geometry is a fascinating possibility which can be used to foster interactions and build smart nanostructures for inhibition or inactivate virus replication. Although pristine C_60_ fullerene has very low solubility in water, it can form a stable colloidal solution^30^, containing both single C_60_ molecules (0.72 nm) and their spherical-like aggregates, the size of which is comparable to the average particle size of SARS-CoV-2—about 120 nm in diameter^31^. The mechanism of C_60_ fullerene dispersal in aqueous solutions might be explained by a formation of a covalent bond between the hydroxyls and carbons in the C_60_ molecule cage, as a result of ultrasound treatment that culminates in a consequent easy C_60_ fullerene dissolution^32^*.* Due to hydrophobicity, C_60_ fullerene easily penetrates the biological membrane by passive diffusion or endocytosis^33,34^. C_60_ fullerene serves as an enzyme inhibitor, drug delivery vector, contrast agent for MRT and photodynamic therapy^33,35–37^. Due to the presence of double electron-deficient chemical bonds in the structure, C_60_ fullerene easily attaches free radicals, i.e. is a powerful antioxidant^38–40^, able to effectively exhibit the anti-inflammatory, antibacterial, antitumor, neuro- and radioprotective effects in the in vitro and in vivo systems^35,36,41,42^. Finally, importantly, C_60_ fullerenes and their water-soluble derivatives exhibit remarkable antiviral activity^27,28,35,36^.

Thus, in this work we first analyzed available 3CLpro and RdRp structures and their possibility to interact with C_60_ fullerene employing computational methods. For this purpose, C_60_ fullerene was docked into 3CLpro and RdRp according to obtained binding models (in the case of 3CLpro, it is blocking of a catalytic dyad, for RdRp—blocking of RNA synthesis pore and preventing of binding with Nsp8). Then the molecular dynamics (MD) simulation was performed on obtained “C_60_ fullerene-3CLpro or RdRp” complexes. Subsequently, received MD trajectories were a subject of MMPBSA (molecular mechanics Poisson–Boltzmann surface area) as well as MMGBSA (molecular mechanics-generalized Born surface area) free-energy analysis. We believe that our computational results shed light on possible ways of 3CLpro and RdRp inhibition by nanobiomaterials, reveal the main flexibility properties of investigated targets and indicate favorable and unfavorable amino acid for interaction in selected binding pockets.

## Calculation methods

### Construction of “C_60_ fullerene-3CLpro or RdRp” systems

According to available structure data^43,44^, the X-ray structures of monomeric 3CLpro protein (Protein Data Bank (PDB) ID 6M2N)^45^ and RdRp protein (PDB ID 7BV2)^44^ were retrieved from the RCSB PDB^42^. Firstly, all routine water molecules and native ligands were removed from the protein structure. Then prior to identifying binding pockets the protein structure treatment was done by the addition of missing hydrogen, correcting protonation states of amides, repairing side chains, and in the end, energy minimized. To run molecular docking simulation, the possible binding pockets for C_60_ fullerene were defined by Caver software cavity computational algorithm^46^ and based on literature analysis^14,21,45,47–50^. As a result, three and one possible binding pockets were identified for RdRp and 3CLpro, respectively.

To each target molecular docking was carried out utilizing flexible C_60_ fullerene molecule and rigid 3CLpro or RdRp molecule. The systematic docking algorithm was used (SDOCK+)^51^, implemented in the QXP package^52^ (the method demonstrates all possible conformations of the studied structures with a minimum RMSD (root-mean-square deviation) value^51^). The maximum number of SDOCK routine steps was set to 300, and the 10 best complexes based on the built-in QXP scoring function^52^ were selected for analysis in the next stages of the investigation. The optimal structure of the studied “C_60_ fullerene—3CLpro or RdRp” complexes was determined by the following basic criteria: (1) the area of the contact surfaces of the protein and ligand; (2) the distance between the 3CLpro or RdRp and C_60_ fullerene; (3) the energy characteristics of the binding in the formed complex. As a result of molecular docking to each study one (best) “C_60_ fullerene-3CLpro or RdRp” complex was selected.

### MD simulation protocol

To estimate stability and crucial interactions of obtained complexes after molecular docking, MD simulation was performed. The calculations were done using Gromacs^53^ 5.1.3 in force field^54^ Charmm36. All exploring 3CLpro or RdRp were protonated according to the build-in function in Gromacs 5.1.3. The topology for C_60_ fullerene was generated by SwissParam^55^. The complexes obtained after molecular docking were used for MD simulation. Each system was placed into the center of a periodic cubic box which was then filled with TIP3P water molecules. A minimum 0.9 nm distance was maintained between the nearest atom of the complex and the edge of the simulation box so that the complex can fully immerse with water and rotate freely. Then, to neutralize the system and mimic the cellular environment (pH = 7), Na^+^ and Cl^−^ ions were added to bring the ionic concentration to 150 mM. Here, the solvent molecules are replaced with monoatomic ions, randomly. Next, the obtained complex was energy minimized what also relieved any steric clashes. The system was relaxed by applying the steepest descent algorithm (the maximum number of steps was 50,000). Then the equilibration was computed in two stages: NVT was first equalized at 100 ps, with the second NPT equalization of 1 ns. After that, we launched MD simulation within 50 ns.

Note, that MD simulation was performed 3 times to each investigated “C_60_ fullerene—3CLpro or RdRp” complex. All calculations were done at the temperature of 300 K and at constant atmospheric pressure.

### Binding free energy calculations

The binding energies of each complex were calculated by applying the MMPBSA method of g_mmpbsa toll^56^ according to the following equation^57,58^

$$ {\text{G}}_{{{\text{binding}}}} = {\text{ G}}_{{{\text{complex}}}} - \, \left( {{\text{G}}_{{{\text{protein}}}} + {\text{ G}}_{{{\text{ligand}}}} } \right), $$

where G_complex_: total free energy of “C_60_ fullerene-3CLpro or RdRp” complex, G_protein/ligand_: total free energies of the isolated protein and ligand in a solvent, respectively. And then each parameter was estimated as follows

$$ \begin{aligned} & {\text{G}}_{{\text{x}}} = {\text{ G}}_{{{\text{mm}}}} {-}{\text{ T}}\Delta {\text{S }} + {\text{ G}}_{{{\text{solvatation}}}} , \\ & {\text{G}}_{{{\text{mm}}}} = {\text{ E}}_{{{\text{bonded}}}} + {\text{ E}}_{{{\text{nonbonded}}}} , \\ & {\text{G}}_{{{\text{solvatation}}}} = {\text{ G}}_{{{\text{polar}}}} + {\text{ G}}_{{{\text{nonpolar}}}} , \\ \end{aligned} $$

where x—“C_60_ fullerene-3CLpro or RdRp” complex or just separate protein or C_60_ fullerene, G_mm_—average molecular mechanics potential energy in a vacuum (comprise energy of bonded (E_bonded_) and nonbonded (E_nonbonded_ = E_vdW_ + E_elect_, where E_vdW_—van der Waals energy and E_elect_—electrostatic energy) interactions and calculated based on the molecular mechanics force-field parameters)^59–61^, TΔS—entropic contribution to the system, G_solvatation_—the solvatation free energy (energy required to transfer a solute from vacuum into the solvent).

So, here the Poisson–Boltzmann (PB) equation has solved to estimate polar desolvation energy (G_polar_)^62^. In the PB calculations the grid size was 0.5 Å. The solvent dielectric constant and solute dielectric constant value was 80 and 2, respectively. Nonpolar (G_nonpolar_) contribution was obtained based on solvent accessible surface area (SASA, Å^2^)

$$ {\text{G}}_{{{\text{nonpolar}}}} = \gamma {\text{SASA}} + {\text{b}}, $$

where γ—surface tension of the solvent and b—fitting parameter^63^.

Next MMGBSA technique was used to obtain more precise picture of “C_60_ fullerene-3CLpro or RdRp” interaction^64,65^. The essential idea of MMGBSA is the estimation of binding energy between each residue of 3CLpro or RdRp and C_60_ fullerene. The algorithm of the MMGBSA method is the same as in MMPBSA and presented above. The whole energy parameters were calculated by utilizing all snapshots from the MD simulation trajectory of length 50 ns.

## Result and discussion

### Binding pocket determination

**Figure 1:**
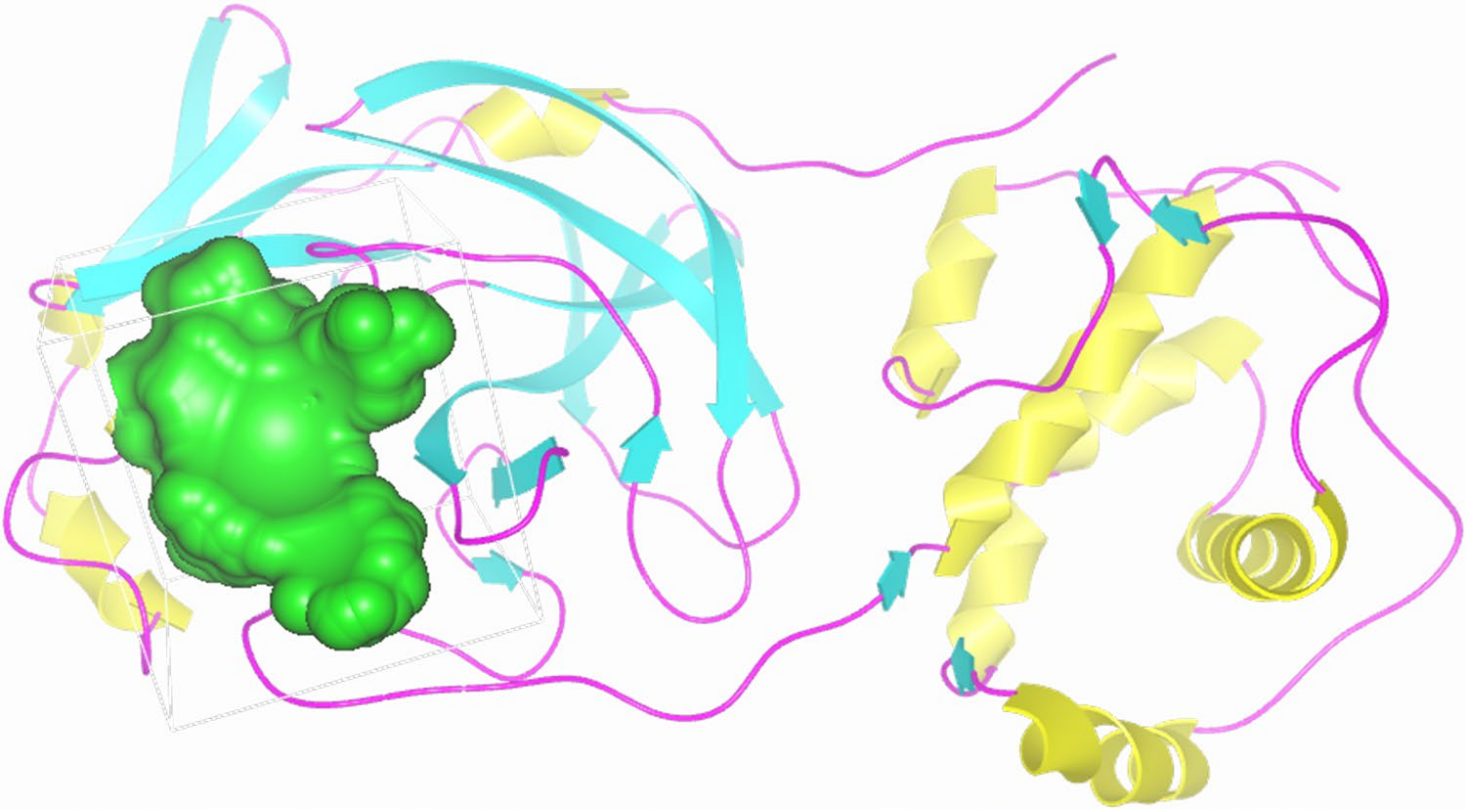
3CLpro secondary structures and potential binding pocket for C_60_ fullerene (blue).

According to the surface analysis of 6M2N and available literature data^21,22,66–68^, the only binding pocket capable of interacting with C_60_ fullerene was identified (Fig. 1).

It is a catalytic binding pocket. This pocket occupies volume 892 Å^3^ and contains amino acids (Met 165, Met 49, Tyr 54, Cys 44, His 41, and Cys 145), which are able to create stacking interactions with C_60_ fullerene. Furthermore, taking into account that this binding pocket directly mediate the mutation of different Nsp^21,69,70^, which are crucial for the virus life cycle, it make 3CLpro a perspective target for drug development against SARS-CoV-2^17,21,22^.

**Figure 2:**
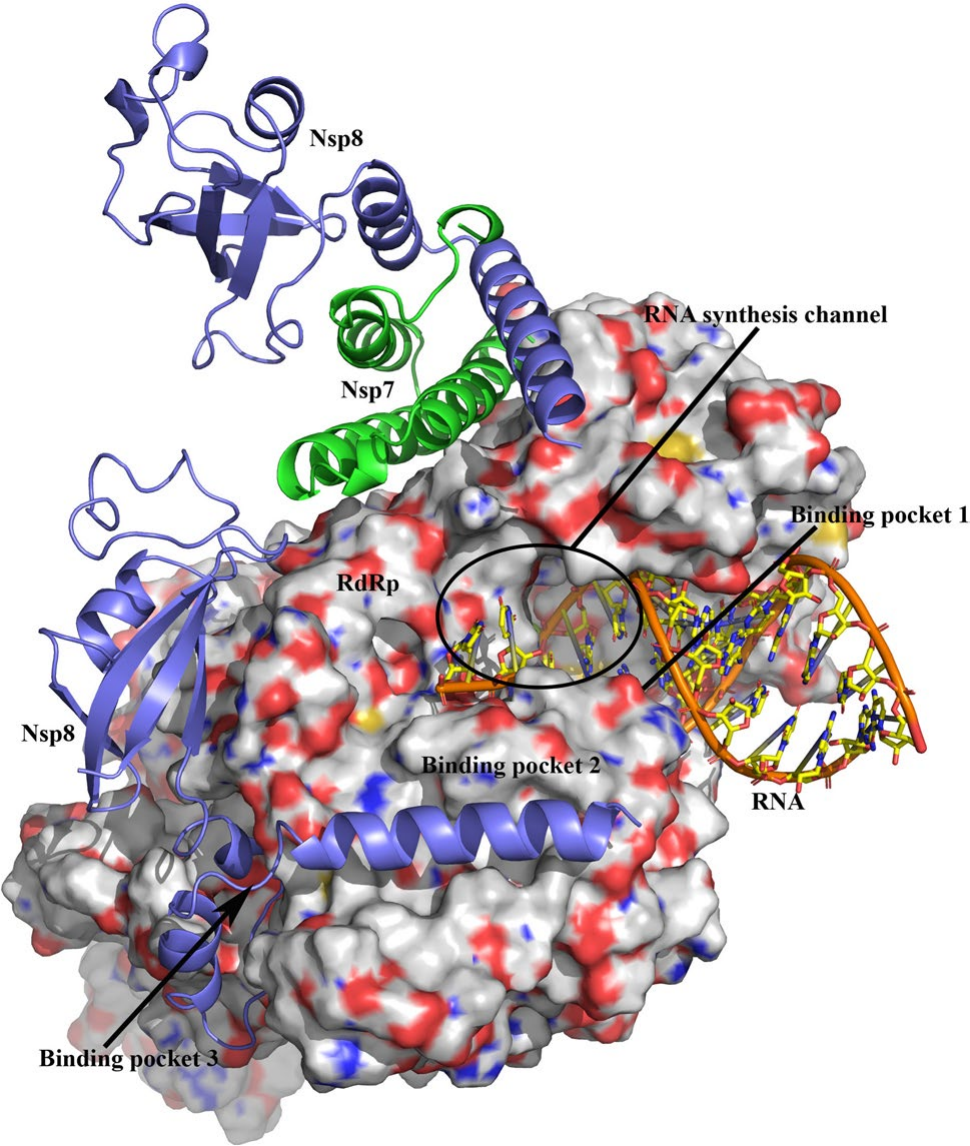
Structure of SARS-CoV 2 RdRp (surface presentation) in complex with Nsp7 (in green), Nsp8 (in purple) co-factors and RNA molecule (stick model). In one case RdRp directly interacts with Nsp8, in other it binds to heterodimer of Nsp7 and Nsp8. Pocket 1 locates in RNA synthesis channel, pockets 2 and 3 locate in direct binding interface between RdRp and Nsp8.

As a result of surface and literature investigation of RdRp, three binding pockets were found. It is well known that RdRp plays a key role in SARS-CoV-2 RNA synthesis^14^. Based on our result two binding models between C_60_ fullerene and RdRp were indicated: model 1–C_60_ fullerene binding in RNA synthesis channel and model 2—simultaneous binding of C_60_ fullerene with pocket 2 and 3 (Fig. 2). So, by blocking the RNA synthesis channel the RNA synthesis procedure will be impossible^19,21,71^. That is why the first detected binding pocket locates in the RNA synthesis channel (Fig. 2). On the other hand, without the assistance of Nsp7 and Nsp8 as co-factors RdRp is not able to carry out its initial functions^14,72,73^. Based on these two other pockets were found in the RdRp-Nsp8 binding interface (Fig. 2). All found binding pockets comprise at least two amino acids, which can create any stacking interactions with C_60_ fullerene. For example, pocket 1 contains Arg 570, Lys 578 and Tyr 690.

### Molecular docking

**Figure 3:**
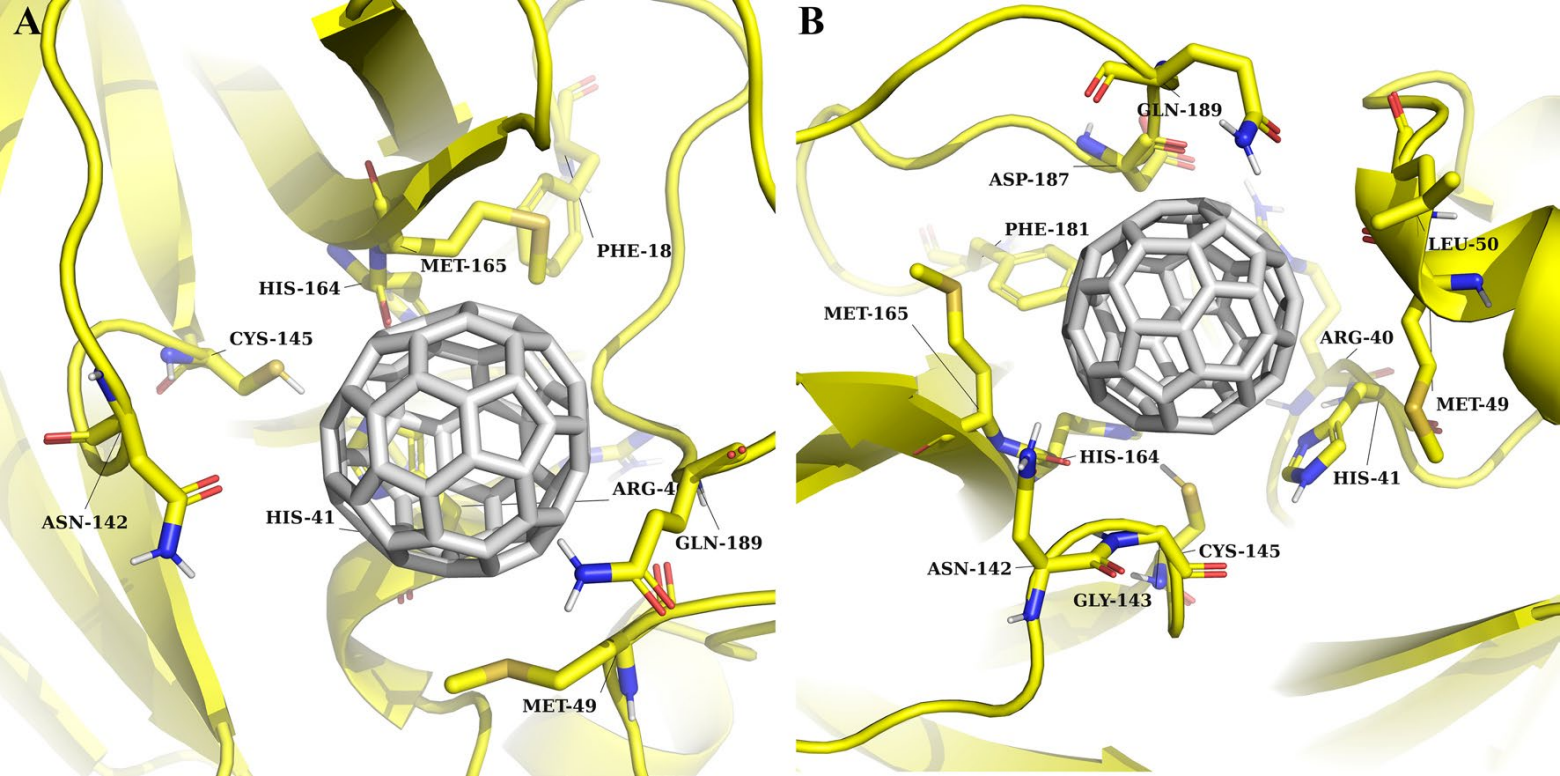
Catalytic binding pocket of 3CLpro: 3CLpro highlighted in yellow, and C_60_ fullerene in grey. Molecular docking result (**A**); MD simulation result (**B**).

*3CLpro* in the presented binding model, C_60_ fullerene is tightly stuck in the catalytic binding pocket. It results in catalytic dyad shielding from interacting with any other molecular structures. There, C_60_ fullerene lies above catalytic His 41 and creates direct stacking interaction with one. Moreover, in this part of 3CLpro catalytic binding pocket C_60_ fullerene creates stacking interaction with Cys 145, Met 49, Met 165, Met 49 and steric interactions with Gln 189 and Asn 142 (Fig. 3A). More importantly, those amino acids are located on different sides of the catalytic binding pocket, and as an outcome, they clamp C_60_ fullerene in the catalytic binding pocket.

**Figure 4:**
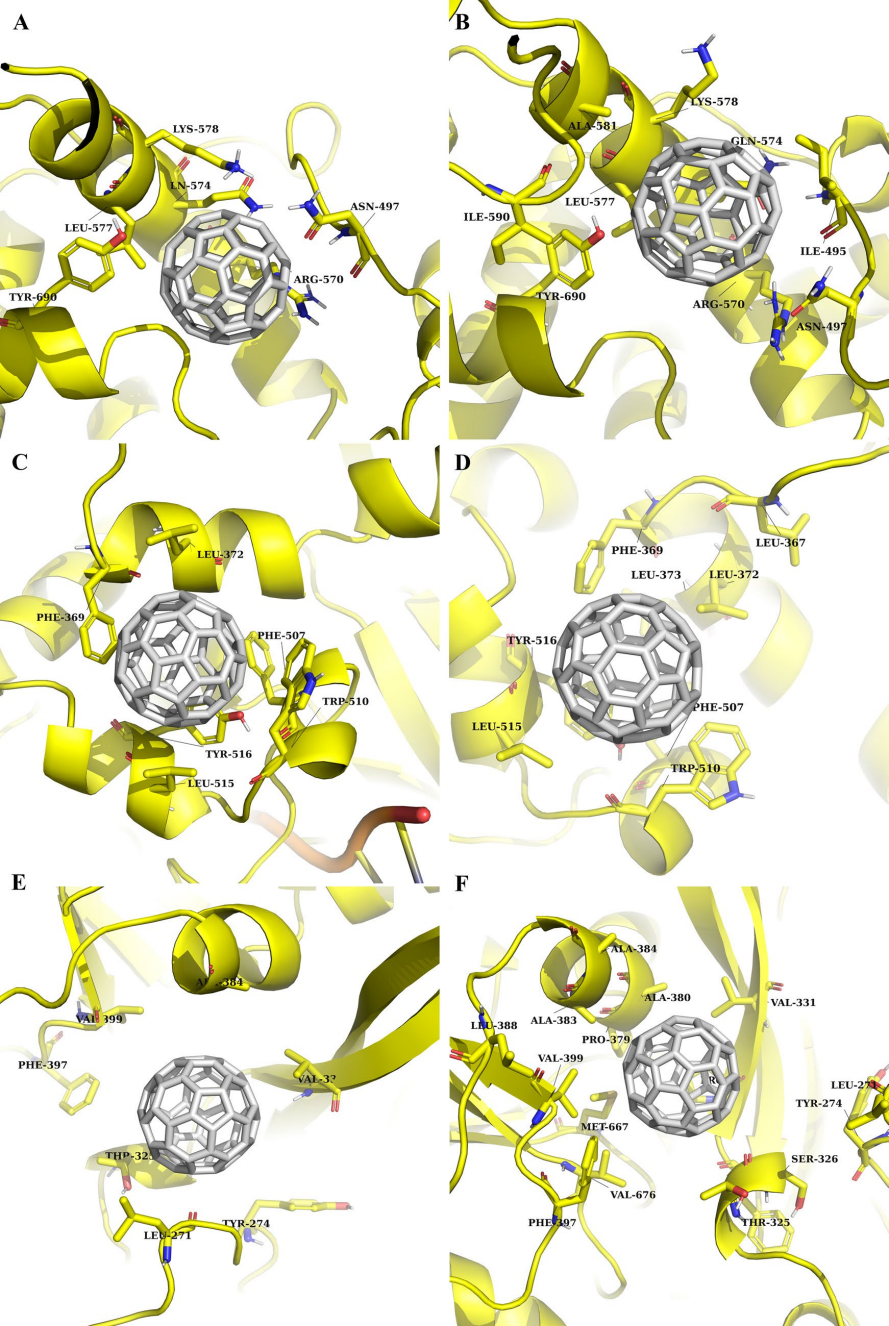
Molecular docking (**A**, **C**, **E**) and MD simulation (**B**, **D**, **F**) results of complex between C_60_ fullerene (in grey) and investigated pockets of RdRp (in yellow): (**A**, **B**)—pocket 1; (**C**, **D**)—pocket 2; (**E**, **F**)—pocket 3.

*RdRp* as with 3CLpro, C_60_ fullerene filled in selected binding pockets of RdRp and tightly clamped there by different stacking and steric interactions (Fig. 4A,C,E). The inhibition of pocket 1 could cause blocking of RNA synthesis channel (Figs. 2, 4A). Here, C_60_ fullerene fits perfectly to the binding pocket and makes π-cation interactions with Arg 570 and Lys 578, T-stacking with Tyr 690, and steric interactions with Asn 497 and Leu 577. Conversely, the C_60_ fullerene interaction with pocket 2 or 3 depicts the model which prevents complex formation between RdRp and Nsp8 (Figs. 2, 4C,E). As a result, RdRp is not able to carry out its initial functions. So, in pocket 2 the bottom part of C_60_ fullerene stacks between Trp 510, Phe 369 and Leu 372, Leu 515 by stacking and steric interactions, respectively. Additionally, Tyr 516 and Phe 507 are located at the bottom of the binding pocket (Fig. 4C). These two amino acids possibly are capable of holding C_60_ fullerene in the current position by stacking interaction. In spite of mentioned above the stability of the obtained complex is questionable because the binding pocket itself isn’t deep. Because of such a flat surface geometry of pocket 2, C_60_ fullerene could be forced out of this binding pocket. Despite the fact that in pocket 3 almost not presents any aromatic amino acid which is able to create stacking interactions with C_60_ fullerene, we think that pocket 3 is promising because of its depth. Here, C_60_ fullerene creates steric interactions with Ala 384, Val 331, Val 399, Thr 325, and Leu 271. Also, the binding pocket contains Phe 397 and Tyr 274, which are spatially close to docked C_60_ fullerene. So, there is a possibility of stacking interaction with those amino acids.

The molecular docking results suggested that in all selected binding pockets C_60_ fullerene is able to create a stable complex with 3Clpro and RdRp targets. The binding with 3CLpro is characterized by one possible binding model. For the RdRp, model 1 (interaction in pocket 1) and model 2 (simultaneous interaction with pockets 2 and 3) were investigated.

### MD analysis

**Figure 5:**
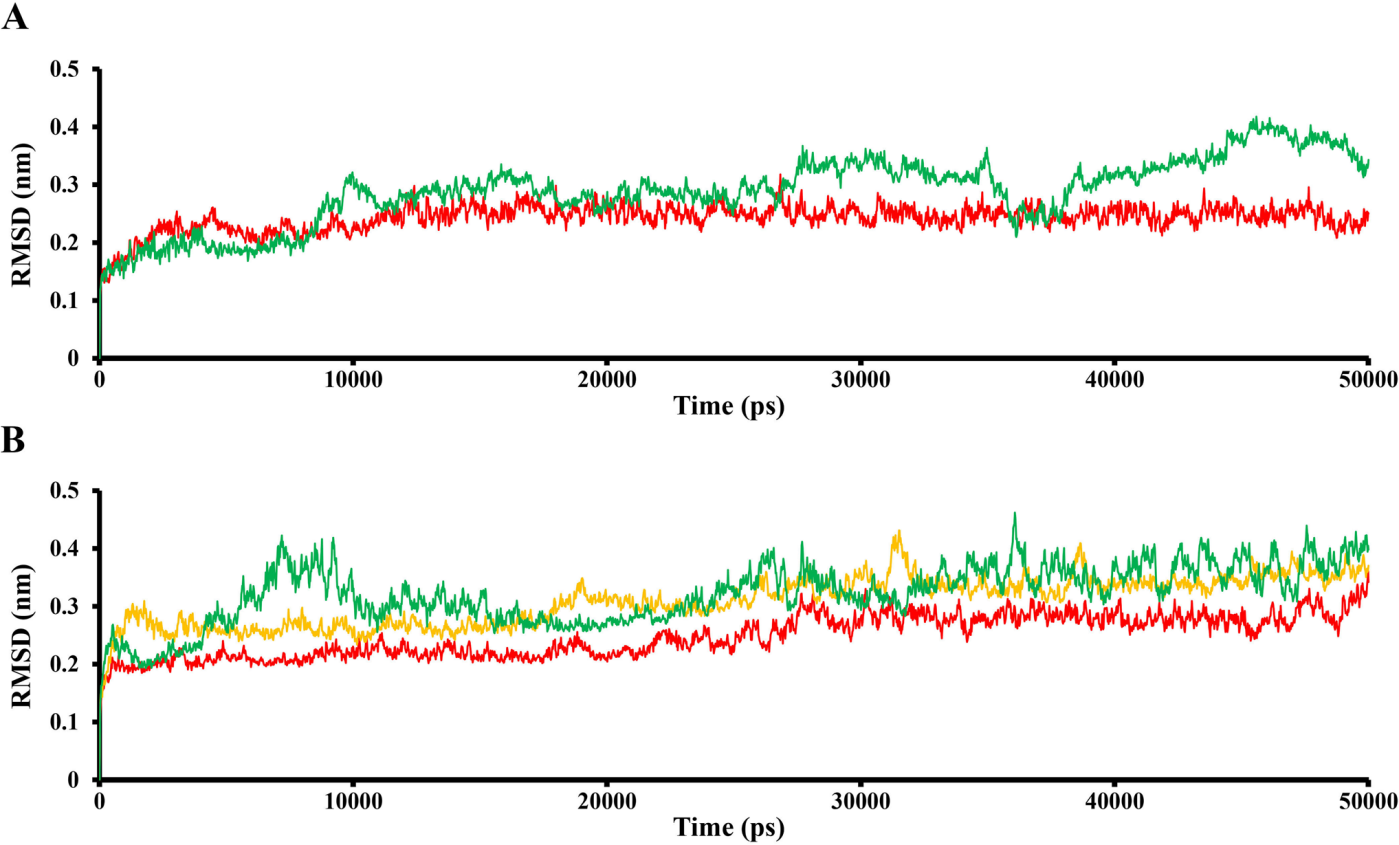
RMSD trajectories of 3CLpro (**A**) and RdRp (**B**) complexes with C_60_ fullerene: 3CLpro free protein molecule—green, “C_60_ fullerene-3CLpro” complex—red, and RdRp free protein molecule—green, “C_60_ fullerene-RdRp” pocket 1 (binding model 1)—red, “C_60_ fullerene-RdRp” pocket 2/3 (binding model 2)—orange.

**Figure 6:**
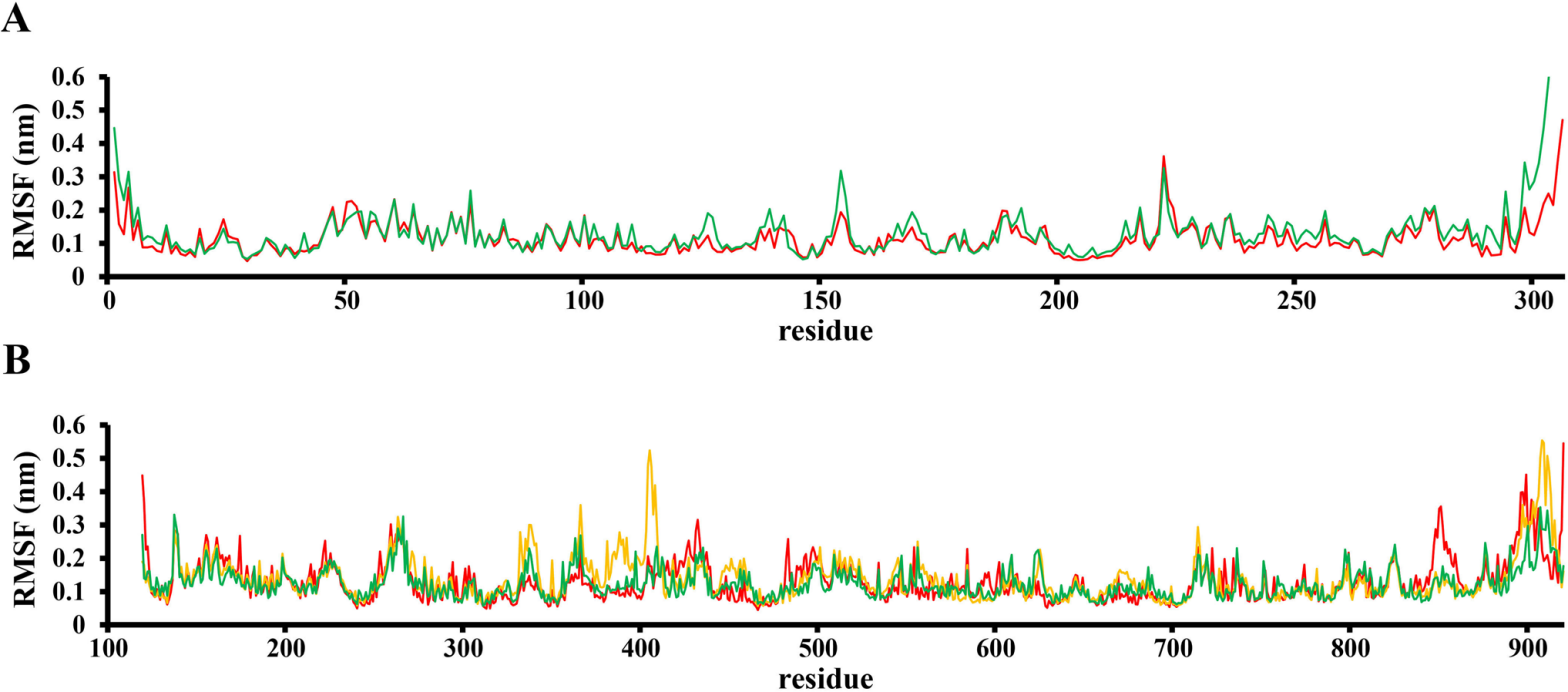
RMSF of the 3CLpro (**A**) and RdRp (**B**) in complex with C_60_ fullerene: 3CLpro free protein molecule—green, “C_60_ fullerene-3CLpro” complex—red, and RdRp free protein molecule—green, “C_60_ fullerene-RdRp” pocket 1 (binding model 1)—red, “C_60_ fullerene-RdRp” pocket 2/3 (binding model 2)—orange.

To obtain more accurate results, 50 ns MD simulations were carried out (Figs. 3, 4, 5, 6). The MD results showed that each investigated “C_60_ fullerene-3CLpro or RdRp” are stable. The RMSD movement during MD simulation of each complex is in a range 2–3 Å (Fig. 5). Furthermore, in some examples C_60_ fullerene is able to form new and more profitable interactions.

*3CLpro* the simulated complex is shown in Fig. 3B. For 3CLpro, it was observed that C_60_ fullerene shifts by 3.2 Å and caused Asn 142 displacement for 4.8 Å toward C_60_ fullerene. In contrast, C_60_ fullerene forced out Gln 189 for 1.8 Å from the catalytic binding pocket. The binding with amino acids like Met 156, Phe 181, His 164, and others are without any fundamental changes. The more intriguing is that both His 41 and Cys 145 (catalytic dyad) are forced out from their initial position by C_60_ fullerene, either. As a result, the integrity of the catalytic dyad is violated and without any doubt, it has a negative impact on 3CLpro functionality. The “C_60_ fullerene-3CLpro” complex was then subjected to RMSF (root-mean-squared-fluctuation) investigation. No, any extreme fluctuations compare to the free form of the 3CLpro molecule were detected (Fig. 6A). For example, the flexibility of His 41 in free and bond to C_60_ fullerene form are 0.8 and 1.4 Å, respectively. Moreover, in the case of the “C_60_ fullerene-3CLpro” complex, the reduction of fluctuation values was determined.

*RdRp* according to the MD simulation result in a case of pockets 1 and 2 the fluctuations of C_60_ fullerene inside both binding pockets (4.1 Å and 3.5 Å, respectively) without significant changes in obtained complexes (Fig. 4B,D) were observed. And, in pocket 3 opposite picture was detected. Here, C_60_ fullerene immersed inside the binding pocket by 4.0 Å. Anyway, the key interactions between C_60_ fullerene and RdRp in whole models remain (Fig. 4B,D,F). So, almost no changes were indicated during MD simulation for pocket 1. Here, it is possible to say that amino acids Ile 590, Tyr 690, Leu 577, and Gln 574 are rigid and just slightly change their initial position during MD simulation (< 0.75 Å), those amino acids interact with C_60_ fullerene by strong steric interactions. Contrarily, Lys 578 and Arg 570 are more flexible and characterized by some displacement (1.5 Å and 1.6 Å, respectively). Interestingly, that the π-cation interaction with Lys 578 isn’t stable, but with Arg 570 is stable. Arg 570 is tightly locked to C_60_ fullerene. Such difference in the interactions between C_60_ fullerene and Lys 578/Arg 570 could be related with the location of those amino acids in the binding pocket. The Lys 578 locates on the edge of the pocket and can freely move (especially, because of Lys amino acid long linker) in contrast Arg 570 locates inside the binding pocket and has less space for movement. In pocket 2 (Fig. 4E) C_60_ fullerene stuck in the insight the pocket, creating steric interaction with Leu 372, Leu 515, and stacking with Phe 369 and Trp 510. Notably, during the whole MD simulation trajectory, the displacement of key binding amino acids (e.g. Phe 369, Trp 510, Tyr 516, Leu 315 and Leu 367) is minimal, about 1 Å. Also, we think that in this position C_60_ fullerene is held by stacking interaction with Tyr 516, which locates in the bottom of the binding pocket 2. Thus, the distance between C_60_ fullerene and Tyr 516 is about 3 Å during all MD simulation. Finally, in pocket 3 inverted picture compare to the above has observed (Fig. 4F). C_60_ fullerene shifted inside the binding pocket and tightly stacked among the surrounding hydrophobic amino acids by interacting with RdRp via stacking and steric interactions with Phe 397 and Val 399, Ala 384, Val 331, Thr 325, respectively. Those interactions become possible due to the shift of Phe 397 (4.9 Å), Val 399 (3.5 Å), and Ala 384 (3.2 Å). Other amino acids Val 331 and Thr 235 located in pocket 3 almost not shifted (about 0.5 Å). As for the previous target, here RMSF analysis has been done as well (Fig. 6B). So, the amino acids 324–342 and 364–410 in the case of C_60_ fullerene binding in pockets 2 and 3 are more flexible compare to the model, where C_60_ fullerene bond to pocket 1 and to unbound RdRp. It is not surprising and could be simply explained by shifting and immersion of C_60_ fullerene in the binding pockets 2 and 3. Of course, during the shifting/immersion C_60_ fullerene in some level pushes amino acids, and it resulted in their mobility. However, in a model with C_60_ fullerene bond pocket 1 mirrored situation is observed. Here, the flexibility of amino acids 840–862 is greater compare to their flexibility in the case of unbound RdRp or bond to pocket 2 and 3. Such a result was obtained due to the fact that in the case of C_60_ fullerene binding in pocket 1 RNA molecule isn't present in RNA synthesis pore (there isn’t enough space for RNA molecule).

### MMPBSA approach

The potential binding affinities within “C_60_ fullerene-3CLpro or RdRp” complexes were estimated by the MMPBSA analysis. Table 1 suggests that the G_binding_ in “C_60_ fullerene-3CLpro or RdRp pocket 3” complexes are more favorable compare to “C_60_ fullerene-RdRp pocket 1/2”. Next, for a better understanding of which energy type has a bigger contribution to complex formation and stability, each separate energy component was analyzed. From Table 1 it can be found that E_vdW_ has the largest contribution to the binding energy of the C_60_ fullerene with 3CLpro and RdRp. As previously, the E_vdW_ values of “C_60_ fullerene-3CLpro or RdRp pocket 3” complexes are far better compare to “C_60_ fullerene-RdRp pocket 1/2”. The other calculated energies (E_elect_, G_polar_ and G_nonpolar_) are positive or close to null. That is why their impact on complex formation and especially stability is unfavorable. However, the E_elect_ and G_nonpolar_ contributions are lightly favorably than G_polar_. Anyway, this slightly bigger effect of E_elect_ and G_nonpolar_ is not much more noticeable in comparison with G_polar_.

**Table. 1:** The energetic parameters obtained from MMPBSA investigation.

Contribution (kJ/mol)	3CLPro	RdRp		
		Pocket 1	Pocket 2	Pocket 3
E_vdW_	− 204.275 ± 18.0	− 164.9 ± 16.7	− 126.3 ± 20.9	− 178.9 ± 13.4
E_elect_	− 0.016 ± 1.4	0.08 ± 2.3	− 0.185 ± 1.15	0.007 ± 1.951
G_polar_	60.8 ± 9.4	46.1 ± 9.5	21.1 ± 12.8	34.1 ± 4.4
G_nonpolar_	− 15.8 ± 1.4	− 12.8 ± 1.2	− 9.9 ± 1.5	− 13.8 ± 1.2
G_binding_	− 159.3 ± 15.2	− 131.5 ± 15.3	− 115.4 ± 23.8	− 158.5 ± 12.5

### MMGBSA approach

**Figure 7:**
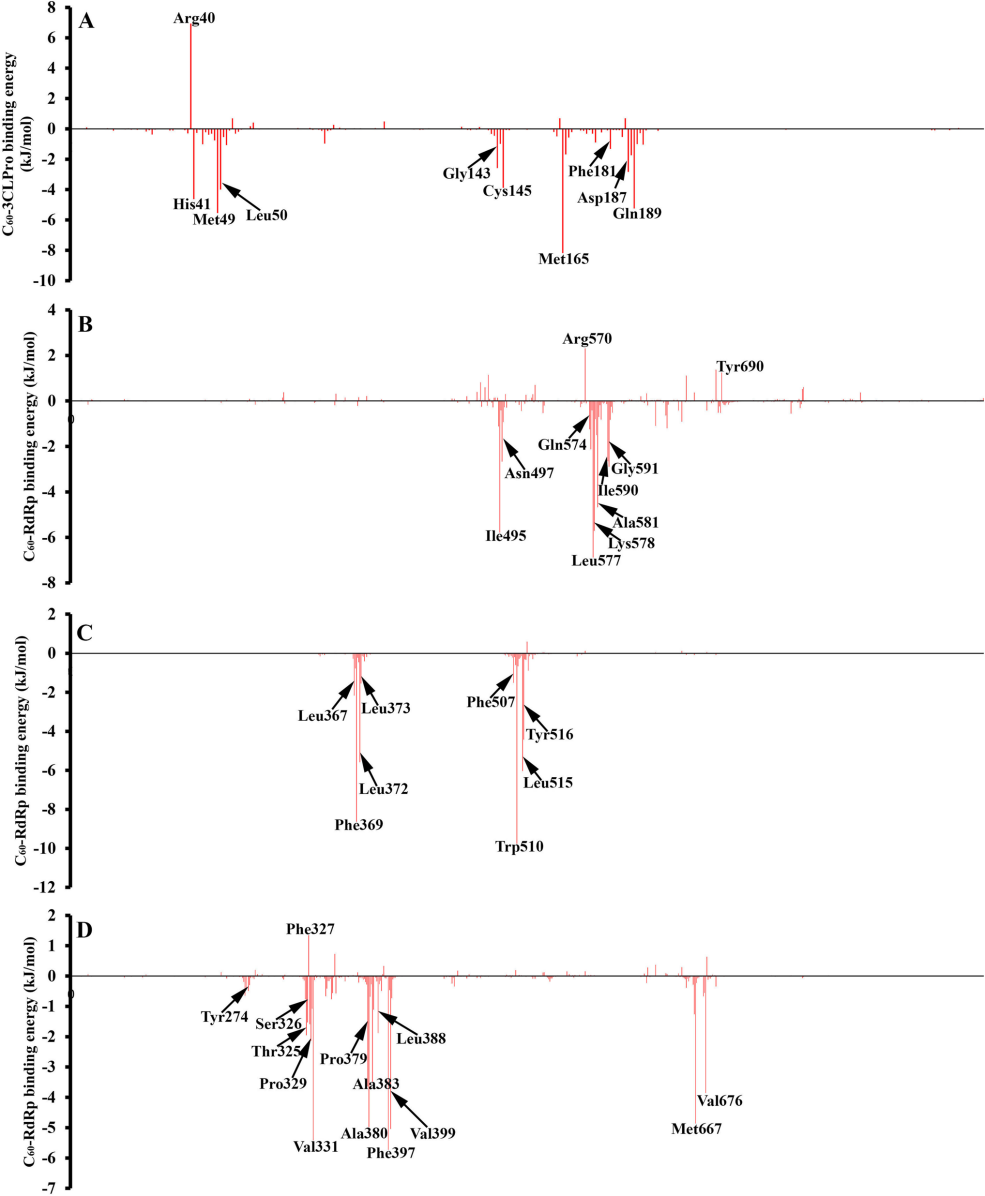
Per-residue binding energy decomposition of investigated complexes: “C_60_ fullerene-3CLpro” (**A**); “C_60_ fullerene-RdRp” pocket 1 (**B**); “C_60_ fullerene-RdRp” pocket 2 (**C**); “C_60_ fullerene-RdRp” pocket 3 (**D**).

To get a more precise binding energy characterization of obtained complexes, a per-residue free-energy decomposition study was performed. According to the results presented in Fig. 7 the most favorable contribution in all models is done by amino acids, which are able to create stacking interaction with C_60_ fullerene. However, some exceptions are detected (e.g. “C_60_ fullerene-RdRp pocket 1”).

*3CLpro* Fig. 7 A emphasizes the role of the catalytic dyad in the binding process. The His 41 and Cys 145 have a positive effect on binding with C_60_ fullerene and the binding energy is -4.63 and -3.88 kJ/mol, respectively. As was mentioned above, due to this, the C_60_ fullerene can immerse into the 3CLpro catalytic binding pocket. Nevertheless, the binding contribution of the catalytic dyad isn’t most favorable. The amino acids Met 49 (binding energy with C_60_ fullerene is − 5.54 kJ/mol), Met 165 (− 8.18 kJ/mol), Leu 50 (-3.99 kJ/mol), Gly 143 (− 2.58 kJ/mol), Asp 187 (− 2.83 kJ/mol) and Gln 189 (− 5.25 kJ/mol) clamp C_60_ fullerene in the binding pocket by stacking and steric interactions (Figs. 3B, 7A). Surprisingly, contrary to the above Arg 40 (6.94 kJ/mol) causes the destabilization of the “C_60_ fullerene–3CLpro” complex formation. This can be explained by the fact C_60_ fullerene prevents the interaction of Arg 40 with water molecules which usually in abundance in any binding pocket.

*RdRp* the comparison of key amino acids in pocket 1 has shown that steric interactions with C_60_ fullerene here are the most favorable (Fig. 7B). Among the whole residue, the interaction between C_60_ fullerene and Ile 49 (− 5.78 kJ/mol), Asn 497 (− 2.67 kJ/mol), Leu 557 (− 6.89 kJ/mol), Lys 578 (− 5.71 kJ/mol), Ala 581 (− 4.68 kJ/mol), Ile 590 (− 2.65 kJ/mol) and Gly 591 (− 2.89 kJ/mol) are far better compared to other amino acids in binding pocket 1. And what is interesting, only one of them forms π-cation interaction (Lys 578) with C_60_ fullerene. Because C_60_ fullerene is an aromatic system and preferably creates different stacking interactions it can cause a negative effect on complex stability. As previously, few amino acids destabilize C_60_ fullerene in binding pocket 1, namely: Arg 570 (2.31 kJ/mol) and Tyr 690 (1.25 kJ/mol). For the binding in pocket 2 stacking interactions are favorable (Figs. 4D, 7C). As follows from the Fig. 7C such amino acids as Leu 367 (− 2.16 kJ/mol), Phe 369 (− 8.64 kJ/mol), Leu 372 (− 5.58 kJ/mol), Leu 373 (- kJ/mol), Phe 507 (− 1.51 kJ/mol), Tpr 510 (− 9.76 kJ/mol), Leu 515 (− 6.04 kJ/mol) and Tyr 516 (− 4.42 kJ/mol) create strong interaction with C_60_ fullerene. Contrary to other investigated binding pockets in this case no amino acid has a huge unfavorable impact on “C_60_ fullerene–RdRp pocket 2” complex formation. From Fig. 7D it can be seen that the interactions between C_60_ fullerene and RdRp pocket 3 are mainly determined by the following residues Ser 326 (− 1.53 kJ/mol), Thr 325 (− 1.95 kJ/mol), Pro 329 (− 2.14 kJ/mol), Val 331 (− 5.42 kJ/mol), Pro 379 (− 3.64 kJ/mol), Ala 380 (− 4.98 kJ/mol), Ala 383 (− 3.48 kJ/mol), Leu 388 (− 1.88 kJ/mol), Phe 397 (− 5.73 kJ/mol), Val 399 (− 5.04 kJ/mol), Met 667 (− 4.86 kJ/mol) and Val 676 (− 3.86 kJ/mol). Here, we can observe the mix of favorable steric and stacking interactions between C_60_ fullerene and pocket 3. For example, the most strongly C_60_ fullerene interacts with Vall 331, Ala 380, Val 339, and Val 676 by steric interactions and despite above by stacking interaction with Pro 379, Phe 397, and Met 667. And finally in this case the C_60_ fullerene interaction with Phe 327 is unfavorable. That can be related to the fact that only the peptide backbone of Phe 327 interacts with C_60_ fullerene. Furthermore, this part of the backbone by C_60_ fullerene isolated from a solvent, what possible is able to cause such tension between those molecular parts/structures.

Summarizing, the in silico approach allows simulating the behavior of C_60_ fullerene in the binding sites of SARS-CoV-2 coronavirus and thus to predict the therapeutic effect of this unique molecule. A complementary interaction of C_60_ fullerene with proteins is a basis of its biomedical effects^74–77^. So, molecular docking and MD simulation were carried out using Toll-like receptors (TLRs play extremely critical roles in maintaining the immune-homeostasis of the human body) including TLR4^75^. The binding of C_60_ fullerene with TLR4 is characterized by complete filling of the hydrophobic pocket of MD-2 domain and the formation of a significant number of stacking interactions (e.g. with Phe 119, Phe 76 and Phe 104). This change of binding site is associated with significant mobility of interacting components: RMSD value for protein is 4.6 Å, and for C_60_ fullerene—5.3 Å. The obtained “C_60_ fullerene-TLR4” complex was characterized by a high energy: − 50 kJ/mol. In a recent study^78^, spike protein of SARS-CoV-2 was found to interact with the extracellular domain of the cell surface TLRs. Intriguingly, the highest binding affinity and strength were evident in the “spike protein-TLR4” complex. Thus, the usage of C_60_ fullerene to inhibit TLR4 as well as 3CLpro and RdRp activation may be an effective strategy to treat COVID-19.

## Conclusion

The computer simulations (docking and molecular dynamics) we presented here suggest that C_60_ fullerene is able to block 3CLpro (blocking of catalytic dyad) and RdRp (blocking model 1 and 2) protein targets of SARS-CoV-2 coronavirus with different mechanisms and suppress its functional activity. The simulations revealed that in all investigated complexes C_60_ fullerene filled in the binding pocket and stuck there by the stacking and steric interactions. Critically that for 3CLpro C_60_ fullerene violated catalytic dyad integrity. All the other changes during simulations there weren’t significant. In the case of RdRp pockets 1 and 2, C_60_ fullerene just fluctuates inside the binding pockets without fundamental changes of previously obtained complexes. The reverse picture has been observed in RdRp pocket 3, here C_60_ fullerene immerses inside the binding pocket and stuck there. The MMPBSA study has shown that in all cases G_binding_ is more favorable in the case of “C_60_ fullerene-3CLpro or RdRp pocket 3” complexes compare to others. And as the main component, E_vdW_ has the biggest contribution to all complexes. Furthermore, the contribution of E_elect_, G_polar_, and G_nonpolar_ are questionable and most of all are unfavorable because of that energies proximity to null or in some cases that energies are far bigger than null. And finally based on MMGBSA investigation favorable and unfavorable amino acids for complex formation with C_60_ fullerene were detected. The results of the study can provide understanding of 3CLpro and RdRp binding with other nanobiomaterials. Moreover, since the pristine C_60_ fullerenes can form a high stable aqueous colloidal solution and exhibit anticoronavirus activity, this expands their use for prophylactic and therapeutic purposes, which requires further in vitro and in vivo testing.
